# Supplementary material for: Unintended consequences of combating desertification in China
Source: Nat Commun. 2023 Feb 28;14:1139. doi: 10.1038/s41467-023-36835-z (PMC9975221; doi:10.1038/s41467-023-36835-z)
Supplement: Supplementary file 1 — Supplementary information [file 41467_2023_36835_MOESM1_ESM.pdf]

Supplementary Information for:

Unintended consequence of combating desertification in China

Contents

Supplementary Note 1 *Desertification-prone region (DPR) in China*

Supplementary Note 2 *"Grain-for-green" and grazing exclusion practices in China*

Supplementary Note 3 *Constructed fractional vegetation coverage (FVC) dataset*

Supplementary Note 4 *Trend of climate change and FVC*

Supplementary Note 5 *Field truthing and interpolation of CNLUCC*

Supplementary Figures 1~15

Supplementary Tables 1~13

Supplementary Note 1 Desertification-prone region (DPR) in China

Desertification is defined as "land degradation in arid, semiarid, and dry subhumid areas resulting from various factors including climatic variations and human activities"[1,2]. From 1999 to the present, every five years, the State Forestry Administration of China (SFAC) has released a bulletin on the status quo of desertification and sandification in China; these bulletins summarize the water erosion, freeze-thaw, salinification, and aeolian desertification statuses of the nation[3]. However, these bulletins do not specify the monitoring criteria, and only the desertification classifications and the corresponding areas in different provinces are released. Based on bulletins released by relevant Chinese agencies, in 2014, the areas affected by water erosion, salinification, freeze-thaw cycles, and aeolian desertification were 25.01×10^4, 17.19×10^4, 36.33×10^4, and 182.63×10^4 km^2, respectively[4].

However, there are differences in the desertification classification schemes applied by different researchers in China and by SFAC. In China, water erosion and freeze-thaw events mainly occur on the Chinese Loess Plateau[5] and the Qinghai-Tibet Plateau[6], respectively. At present, the Chinese Loess Plateau is not regarded as a typical desertification region[7], and under the context of global warming, freeze-thaw events on the Qinghai-Tibet Plateau have positive effects on vegetation growth[8]. Therefore, no severe desertification issues currently exist in either of these regions, and most researchers in China do not regard water erosion, freeze-thaw events or the resulting ecological, environmental and food security issues as consequences of desertification. In addition, although salinification also occurs in parts of arid, semiarid, and semihumid regions and is one form of desertification[9], the reversal of salinification resulting from a decreased groundwater level[10-12] and the limited area affected by salinification ($\sim17\times10^4$ km^2) means that salinification is not considered a serious desertification issue and does not significantly affect the ecological environment or food security in China. Due to the exceptionally large

affected area (~184 ×10^4 km^2, comprising ~90% of the desertification-prone area in China), 'aeolian desertification' is usually regarded as a kind of desertification. This process is formally defined as "the destruction of an ecological balance as a result of excessive human activities under conditions characterized by high wind activity in regions with sandy surface sediments, accompanied by evidence of intensifying wind activity occurring simultaneously with land degradation"[13-15].

Desertification-prone regions are mainly covered by sandy lands, the ecological landscapes of which are similar to those of grasslands and steppe regions, as has been noted in various vegetation classification schemes[16,17] developed for the arid, semiarid and semihumid regions of China. Under the condition that the underlying soils consist mainly of sands (particles of 0.05-2 mm in diameter), these landscapes are referred to as 'sandy lands'; according to the vegetation coverage, these areas can be further classified as mobile, semimobile, semianchored, and anchored sandy lands. The Gobi Desert is defined as "wide, shallow basins with smooth rocky bottoms filled with sand, silt or clay, pebbles or, more often, with gravel"[18]; the land surface of the Gobi Desert is usually covered by a layer of coarse sands or gravels (>0.5 mm in diameter) with very low vegetation coverage (<5%)[19]. If the vegetation coverage or productivity of sandy lands/the Gobi Deserts decreases, desertification occurs. From the historical period to the present, several sandy lands have been reclaimed and have become major grain production areas in China[20-22], and nearly all of the sandy lands have been used for animal grazing[23]. Although the intensity of human activity in the Gobi Desert is extremely low[24], due to the extremely low productivity of this region[25-27], desertification in such areas is usually not considered[17]. According to the above definition, we compiled the spatial distributions of the DPR (Supplementary Fig. 1) and counted the land use types (Supplementary Fig. 6), population, GDP (Supplementary Fig. 11), grain production, livestock production, and related income of individuals (Supplementary Fig. 7) in the DPR. The recent (2015-2019) specific economic situation of the DPR is shown in Supplementary Table 1.

Supplementary Note 2 "Grain-for-green" and grazing exclusion practices in China

Since the late 1970s, and especially from the early 2000s to the present, multiple desertification-combating programmes, mainly including the "Natural Forest Protection", "Great Green Wall", "Returning Farmland to Forest/Grassland", "Pasturing Prohibition", and "Beijing-Tianjin Sandstorm Source Control" programmes, have been launched to combat desertification, control dust storms, and improve the ecological environments in the DPR of China (Supplementary Tables 1, 11). Although diverse countermeasures are employed in each programme, the major countermeasures of these programmes can be roughly divided into two categories: returning farmlands to forests/grasslands and afforestation ("grain-for-green" practices), and grazing exclusion practices. Following the practices of these programmes, the Chinese government invested some money (Supplementary Table 1), a

portion of which was paid to farmers and herders (Supplementary Table 6). In addition, following the government plan, in the near future, “grain-for-green” and grazing exclusion practices are expected to continue (Supplementary Table 2).

However, there are no official details regarding limited or banned regions and land use types covered by the “grain-for-green” and grazing exclusion practices; therefore, we identified these regions and land use types based on multiple-period land use data by referring to the relevant legal literature, and acquired the proportions of DPR areas in each province related to the two practice types (Supplementary Fig. 3). Then, we applied a statistical framework to identify the contributions of climate change and the two practice types to vegetation restoration in the DPR (Supplementary Fig. 9) and calculated the production and income losses induced by the two practice types in the DPR (Supplementary Fig. 4; Supplementary Table 4).

Supplementary Note 3 Construction of the fractional vegetation coverage (FVC) dataset

We used the long-term, continuous NDVI dataset of the Blended Vegetation Health Product (VH NDVI) and 250-m MODIS NDVI data to construct the FVC dataset. The VH NDVI product (for 1982-2018), which has a spatial resolution of 4 km and a temporal resolution of 7 days, was retrieved from the Visible Infrared Imaging Radiometer Suite (VIRS) (available since 2013) and Advanced Very-High-Resolution Radiometer (AVHRR, available from 1982 to 2012) land surface data (ftp://ftp.star.nesdis.noaa.gov/pub/corp/scsb/wguo/data/Blended_VH_4km/VH/). Sixteen-day composite 250-m MODIS NDVI values were derived from the MOD13Q1 Terra Vegetation Indices (https://ladsweb.modaps.eosdis.nasa.gov/missions-and-measurements/products/MOD13Q1), which has been available since 2000.

To match the temporal resolution of the MODIS and VH NDVI datasets, a dataset with a spatial resolution of half a month was composited using the maximum value composite (MVC) method. Before resampling, missing data in the MODIS and VH NDVI dataset were compensated using the nearest value at the temporal scale. When the monthly VH NDVI data is less than 4 periods, the nearest time data is used as substitute. Meanwhile, in order to maintain the continuity of monthly scale data, the MVC was used to generate NDVI for the two periods of the upper and the second half of the month respectively. This work ensures that the two sets of data have 24 periods per year. To construct a high-quality MODIS data map, median filter technology[28,29] was used to smooth the MODIS NDVI profiles. Then, based on the Quality Control (QC) information for MODIS NDVI (http://lpdaac.usgs.gov/modis/qa/mod13a2 _qa_v5.asp), NDVI values derived using the main radiative transfer method ($Q<3$) were retained, while those derived using the empirical method due ($Q\geq3$) to geometric incompatibility or other problems were excluded and replaced by the filtered values. Due to the large spatial heterogeneity of the start and end times of the growing season in the DPR, the accumulated annual NDVI was

used to reflect vegetation growth characteristics. The annual NDVI value was calculated as follows:

$$NDVI_{y,i} = \sum_{m=1}^{m=24} NDVI_{m,i} \quad (1)$$

$$NDVI_{m,i} > 0 \quad (2)$$

where $NDVI_{m,i}$ is the NDVI value of the i_{th} grid point at the *m*th time and $NDVI_{y,i}$ is the annual NDVI value of the corresponding grid point. To match the spatial resolution and timespan of the MODIS dataset in the DPR, annual VH NDVI data for 1982-2018 were resampled to a 250-m spatial resolution using the bilinear interpolation method to provide information about the distribution of vegetation and help reduce the probability of non-vegetation being treated as plants in the constructed NDVI (CD NDVI) datasets. Therefore, based on the NDVI data sources and methods described above, the constructed NDVI dataset (CD NDVI) was created, and the annual value of NDVI value could be calculated as follow:

$$ANDVI_{M,ix,jx} = \frac{\sum_{y-2000}^{y=2018} NDVI_{M,y,ix,jx}}{19} \quad (3)$$

$$ANDVI_{B,ix,jx} = \frac{\sum_{y-2000}^{y=2018} NDVI_{B,y,ix,jx}}{19} \quad (4)$$

$$V_{ix,jx} = \frac{ANDVI_{B,ix,jx}}{ANDVI_{M,ix,jx}} \quad (5)$$

$$NDVI_{ix,jx} = \left(1 + \frac{NDVI_{B,i,j} - ANDVI_{M,ix,jx}}{ANDVI_{M,ix,jx}}\right) \times \frac{NDVI_{B,i,j}}{V_{ix,jx}}, \ i < ix, j < jx \quad (6)$$

$$i = h + ix \cdot \Delta_i, h < \Delta_i \quad (7)$$

$$j = v + jx \cdot \Delta_j, v < \Delta_j \quad (8)$$

where i (or ix) and j (or jx) refer to pixel in the i_{th} (or ix) row and j_{th} (or jx) column in the image data, respectively. Δ_i is $\frac{ix}{i}$; Δ_j is $\frac{jx}{j}$. $NDVI_B$ refers to the VH NDV, $NDVI_M$ refers to the MODIS NDVI, and ANDVI is the annual NDVI value. Then, we reproduced a 250-m-resolution FVC dataset (CD FVC) using the improved pixel bipartite model for the 1982-2018 period (see Methods).

To test the robustness of the CD NDVI and FVC dataset, we used the GIMMS3g NDVI (https://iridl.ldeo.columbia.edu/SOURCES/.NASA/.ARC/.ECOCAST/.GIMMS/.NDVI3g/.v1p0/) and SPOT NDVI (https://land.copernicus.vgt.vito.be/PDF/portal/Application.html#Browse;Root=513186) datasets to construct VH, GIMMS3g, SPOT, and MODIS FVC data series. First, because assessing the consistency of the spatiotemporal variation trends is an effective method for verifying the quality of data from different sources[30,31], we compared the CD NDVI with the VH, GIMMS3g, SPOT, and MODIS NDVI data from 2000-2013. The results show that although, from 2000 to 2013, the average CD NDVI value (1.91±0.15, mean±1 standard deviation) was 0.51 and 0.39 times higher than that of GIMMS3g and SPOT NDVI, respectively, all of the NDVI datasets showed significant increasing trends on the annual scale ($P < 0.01$) (Supplementary Fig. 13). This analysis suggests that the annual variation trends of the five NDVI datasets were relatively consistent in the DPR. Secondly,

an intercomparison of the annual variation trends of the five NDVI datasets in different subregions in the DPR was performed. The results (Supplementary Fig. 14) also show that, at the annual scale for the nine subregions in the DPR, all of the NDVI datasets exhibit increasing trends (with slopes >0), indicating that the variation trend of the CD NDVI is similar to the VH, GIMMS3g, and MODIS NDVI. Third, the zonal variation trends of the CD NDVI and MODIS NDVI from 2000-2018 (Supplementary Fig. 15a, b) also show a significant and consistent decreasing trend over the latitudinal range of 35.5° N - 42.5°N and an increasing trend over the range of 46.5°N - 50°N, suggesting that spatial consistency exists between these two datasets. Finally, the CD NDVI and MODIS NDVI were also highly correlated at both the zonal and pixel levels (R $\geqslant$ 0.879 (P <0.001) and $\geqslant$ 0.564 (P <0.001), respectively) (Supplementary Fig. 15c). Therefore, the CD NDVI dataset accurately quantified the spatial and temporal NDVI variations and was available for the entire DPR region. Using the methods described above, we also verified the reliability of the CD FVC data (Supplementary Fig. 8; Supplementary Table 8; Supplementary Table 9).

Supplementary Note 4 Climate change and FVC trends

The temperature, precipitation, and FVC trends in the DPR of China from 1982-2018 are shown in Supplementary Fig. 5 and Supplementary Table 3. To assess the impacts of future climate and CO_2 change on vegetation restoration in the DPR, we obtained monthly output data from 21 commonly used GCMs spanning the period from 1982-2050 from the Earth System Grid Federation (ESGF, https://esgf-node.llnl.gov/search/cmip6/) (Supplementary Table 10). The time series of CMIP6 model outputs comprise two two parts: 1) historical simulations (1982-2014) used for comparisons with the observed data and 2) ScenarioMIP simulations (2015-2050) that were used to show the future changes in the climate system under different SSP and RCP scenarios [32,33]. The SSP1-2.6, SSP2-4.5, and SSP5-8.5 scenarios are three of the scenarios commonly used in future projection assessments[33], and thus were thus chosen in this work to analyse future FVC changes in the DPR. In addition, only the r1i1p1f1 simulations of the 21 GCMs were used here to ensure consistency among all of the output data[34]. In general, climate variables that may potentially affect vegetation growth include the precipitation, air temperature (mean, maximum, and minimum), solar irradiation, and wind, and therefore the monthly output data of these variables were used in this study.

In addition to climate factors, the fertilization effect of anthropogenic CO_2 increases can also favour vegetation growth and rehabilitation[35,36]; therefore the monthly atmospheric CO_2 concentration data during the historical (1981–2014) and future (2015–2100) periods under different scenarios were also used in this study. These data were generated by the simplified climate carbon cycle model (MagicC7.0)[37] at a longitudinal interval of 0.5° by the University of Melbourne (https://greenhousegases.science.unimelb.edu.au/#!/view).

Differences between GCM simulations and observed data occur because of uncertainties in initial fields, the depictions of physical processes, and the probabilities associated with climate systems. To address these differences and uncertainties, we used the delta change (DC) method modify the GCM simulations by removing the errors between the simulated and observed data[38] during the reference period of 1982–2014 at each weather station and then applied the correction to modify the simulated data during the period of 2015-2050 period:

$$x_f^* = \mu_o + (x_f - \mu_m) \tag{10}$$

$$x_f^* = \mu_o \times (x_f/\mu_m) \tag{11}$$

where the subscript f indicates the future; μ_o and μ_m are the means of the observed and model-simulated data, respectively; and x_f and x^*_f are the original and modified simulation data, respectively. Some of the variables (such as temperature) were modified using Equation 10, while other variables (e.g., precipitation) were modified using Equation 11. Then, we used a multiple model ensemble (MME) based on the average equal-weights GCMs as the input for the simulations to prevent potential errors resulting from using a single GCM.

The temperature and precipitation trends in the DPR of China from 2015 to 2050 were added to Supplementary Fig. 5 to facilitate a clear comparison. Then, we proposed an SMLR model for each pixel based on the reference period of the FVC and environmental factors data (the corresponding coefficient of determination is shown in Supplementary Fig. 10) and simulated the future trends from 2015-2050 under the SSP1-2.6, SSP2-4.5, and SSP5-8.5 scenarios from CMIP6 model experiments (Fig. 5; Supplementary Table 7).

Supplementary Note 5 Field truthing and interpolation of the CNLUCC data

Mistakes in the characterization and positioning of patches due to operator misjudgements are inevitable accidental errors in visual interpretation work. Therefore, data teams have evaluated the accuracy of the reported land use data for each period by randomly sampling or sampling along the lines and comparing the actual and interpreted results at the sample points one-by-one using global positioning system (GPS) data[39]. At present, such validations have covered most regions and each period in China (e.g., Supplementary Table 12 shows the accuracy analysis results for Northeast China) and show that the overall accuracy of the dataset exceeds 90%[40,41]. Therefore, these data have been widely trusted and applied[42,43].

To obtain the arable lands and livestock production per unit area of grasslands and farmlands from the country-level database covering the DPR from 1982-2020, we used CNLUCC data to extract the cultivated land and grassland areas in each county (district). As CNLUCC has only 8 years of data (1980, 1990, 1995, 2000, 2005, 2010, 2015, and 2020), we built a linear regression model based on the available data and used this model to generate a complete time series via interpolation:

$$Y = \alpha + \beta X + \varepsilon \tag{9}$$

where Y is the time series of the cultivated land and grassland areas, X is the year, β is the regression coefficient, and ε is the regression error or residual. A least-squares method was used to fit equation (1) and, subsequently, to estimate the missing data. The goodness of fit was assessed by the coefficient of determination (R^2)[44]. Cross-validation[45] was used to test the performance of the linear interpolation method filling the data gaps. For land use data, the validations showed that the average interpolation errors for 126 counties (representing 48.65% of the total) were within 10%, and 101 counties (39.00% of the total) had average interpolation errors of 10-20% (Supplementary Table 10). Given the classification errors in the land use data[41], the results suggested that the linear interpolation method could reliably estimate the missing data. Table 1 lists the cross-validation results obtained for a randomly selected proportion of counties in 1995, 2005, and 2015. The missing socioeconomic data values are scattered, so the test results are not shown.

Supplementary Figures 1~15

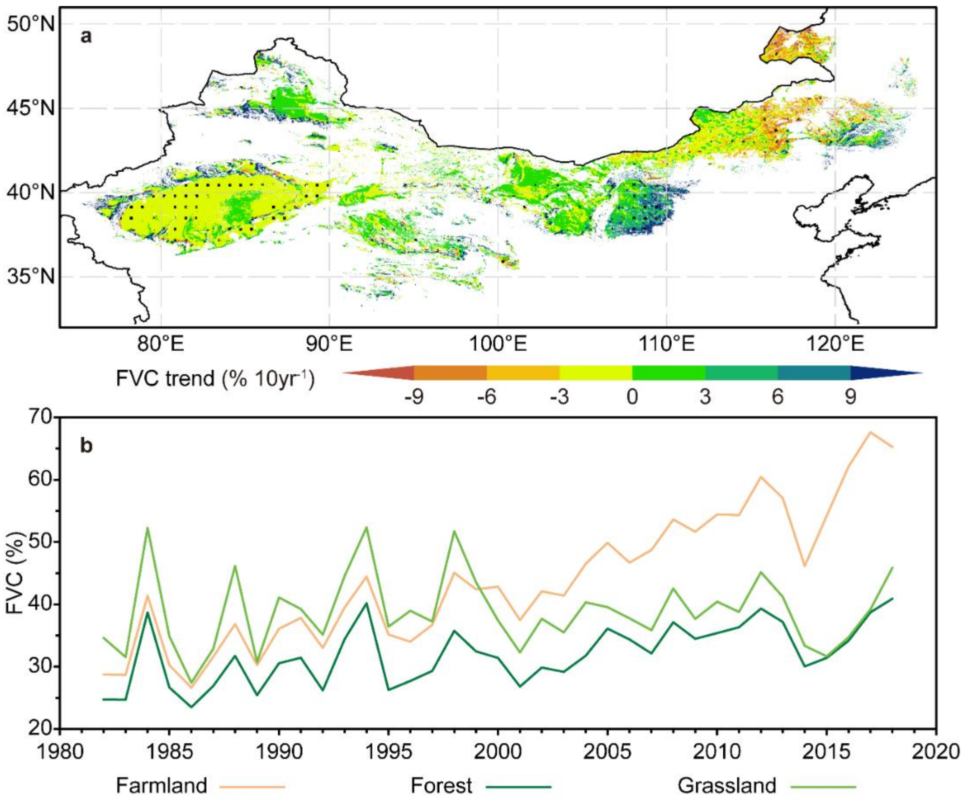

Supplementary Fig. 1 Fractional vegetation cover (FVC) trends in the desertification-prone region of China during the 1982 to 2018. a: Spatial patterns of the linear FVC trends; b: the annual average FVCs in farmlands, forests, and grasslands. The stippling in panel (a) marks regions with significant trends (the Mann-Kendall test passed at the 95% significance level).

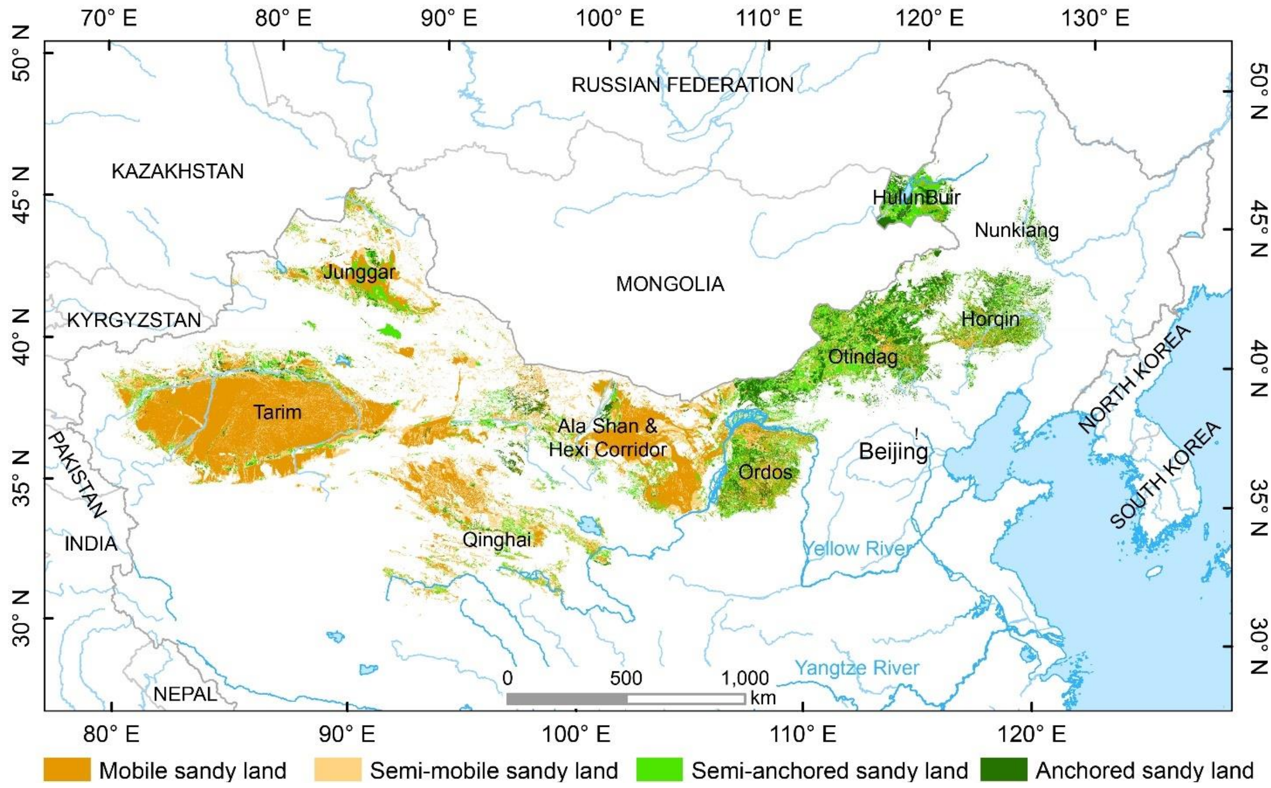

Supplementary Fig. 2 Location and subregions of the desertification-prone region in China.

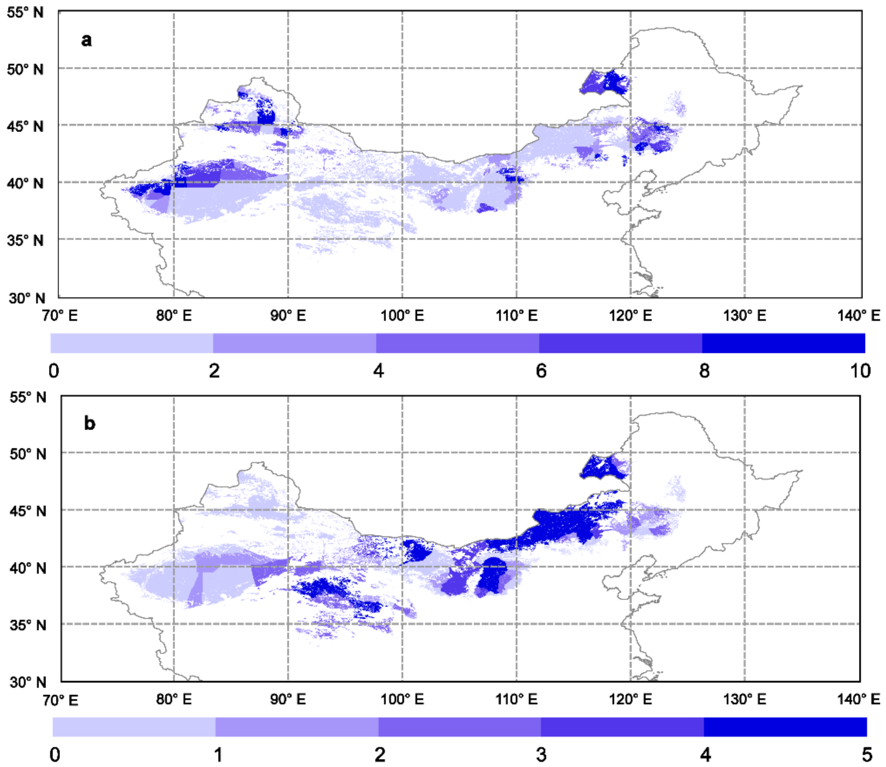

Supplementary Fig. 3 County-level spatial distributions of combating desertification practices in the desertification-prone region since 2000. a: cultivated land areas involved in "grain-for-green" practices (10^3 ha); b: grassland areas involved in grazing exclusion practices (10^{-1} million ha). See the Methods section for more details.

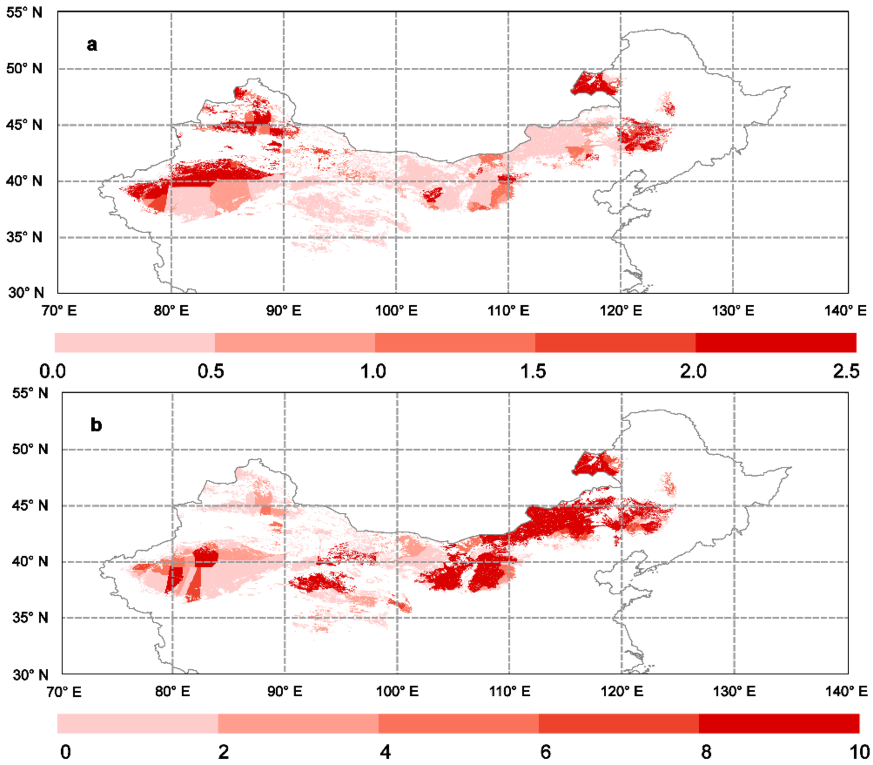

Supplementary Fig. 4 County-level decreases in grain production and livestock production in the desertification-prone region of China since 2000. a: grain production decreases (10^{-3} million tonnes); b: livestock production decreases (10^{-1} million standard sheep units).

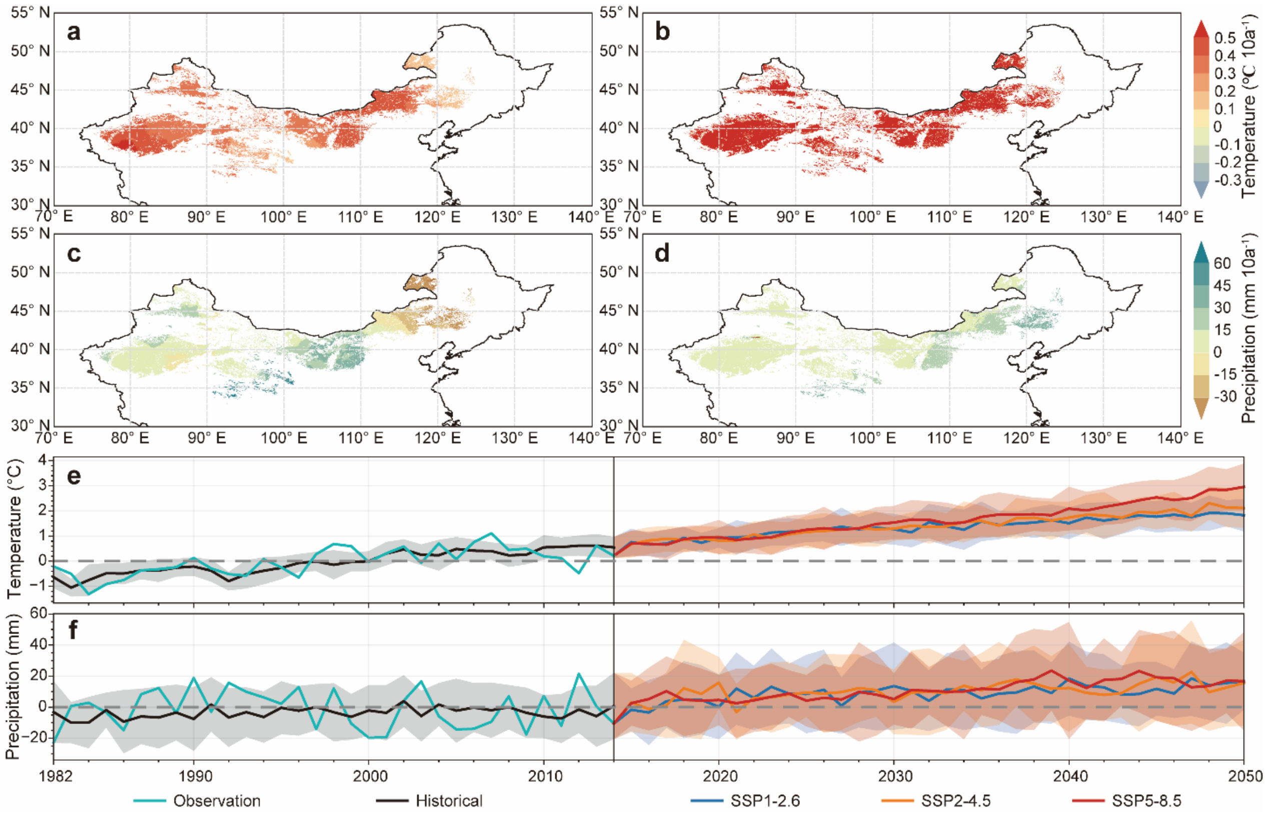

Supplementary Fig. 5 Historical and future climate change trends in the desertification-prone region of China. Spatial distributions of temperature (a) and precipitation (c) trends from 1982 to 2018 and spatial distributions of temperature (b) and precipitation (d) trends from 2015 to 2050. The trend lines from 1982 to 2050 are also provided (e, f). The values in panels (b) and (d) are the average values derived under different shared socioeconomic pathway (SSP) and representative concentration pathway (RCP) scenarios (i.e., SSP1-2.6, SSP2-4.5, and SSP5-8.5) from the Coupled Model Intercomparison Project Phase 6 (CMIP6) model experiments. All scenario simulations were modified by removing the errors between the CMIP6-simulated (the line referred to as Historical in panels (e,f)) and observed (the line referred to as Observation in panels (e,f)) data during the 1982-2014 reference period and the difference between the three scenarios in panels (e,f) shown since 2014. The widths of the bands in panels (e) and (f) indicate the standard deviations among the different global climate models. To compare present and future climate conditions more intuitively, the absolute climate values are replaced by anomaly values obtained based on climatic averages during the 1982 to 2015 period in panels (e) and (f). See Supplementary Note 4 for details about CMIP6 data processing.

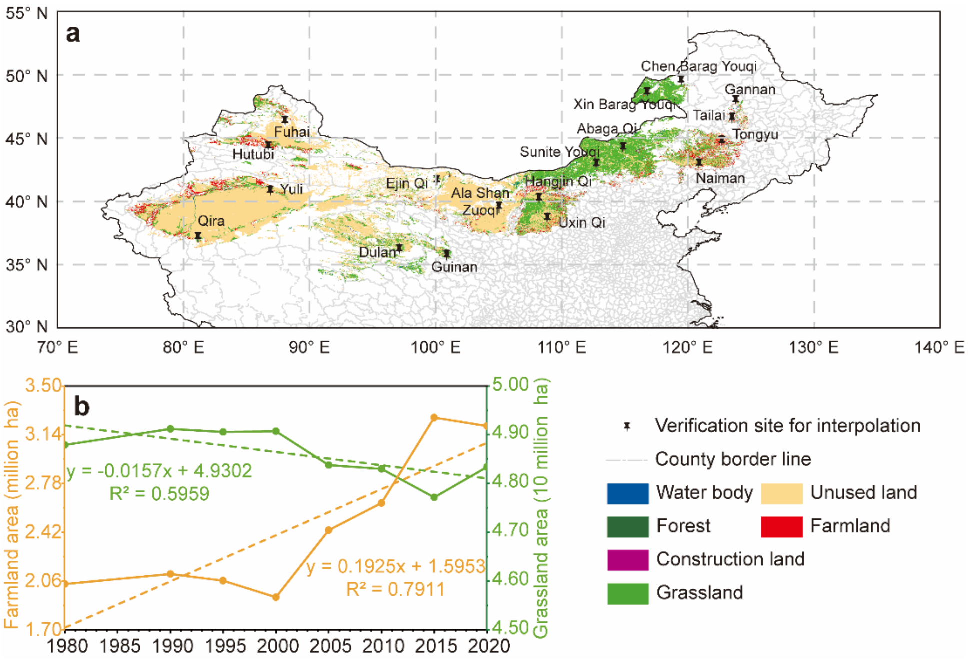

Supplementary Fig. 6 Land use spatial pattern in 2020 (a) and area change trends during 1980 to 2020 (b) in the desertification-prone region. The information representing 1980, 1990, 1995, 2000, 2005, 2010, 2015 and 2020 was provided by the Multi-Period Land Use Land Cover Remote Sensing Monitoring Dataset for China, and the other data were interpolated based on the line trends. See Methods and Supplementary Note 5 for more details.

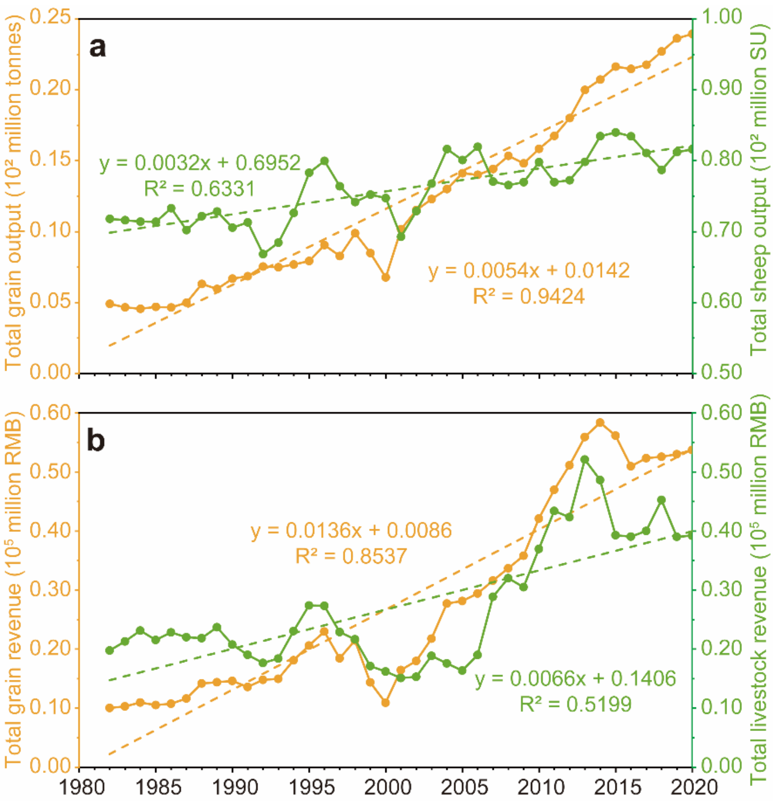

Supplementary Fig. 7 Annual actual yields of grain and animal husbandry (a) and the resulting incomes (b) in the desertification-prone region during 1982-2020. 1 RMB ≈ 0.14 USD.

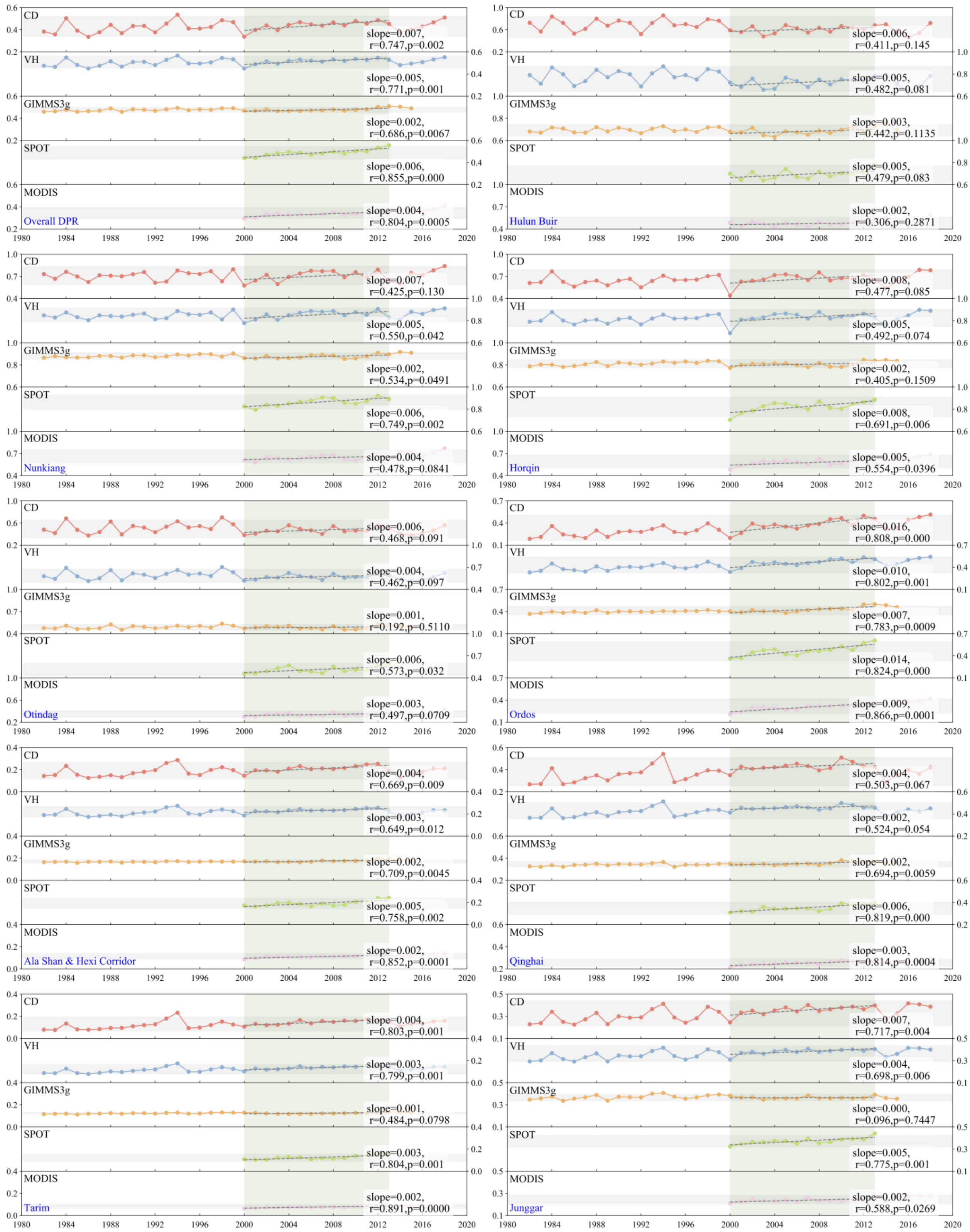

Supplementary Fig. 8 Annual variation of the constructed (CD), the Blended Vegetation Health (VH), Global Inventory Modeling and Mapping Studies (GIMMS3g), Satellite Pour l'Observation de la Terre (SPOT), and Moderate-resolution Imaging Spectroradiometer (MODIS) fractional vegetation cover (FVC) in the desertification-prone region and in each subregion. The locations of subregions are shown in Supplementary Fig. 2. The values are listed as reginal averages for each year. The grey shading represents FVC ± 1.96 standard deviation (FVC is the multiyear mean), and the green shading represents the 2000-2013 period. All statistical degrees of freedom are 13, and the sample sizes are all 14. The r value is Pearson’s R. The five different coloured curves, from top to bottom, correspond to the CD, VH, GIMMS3g, SPOT, and MODIS FVC, respectively.

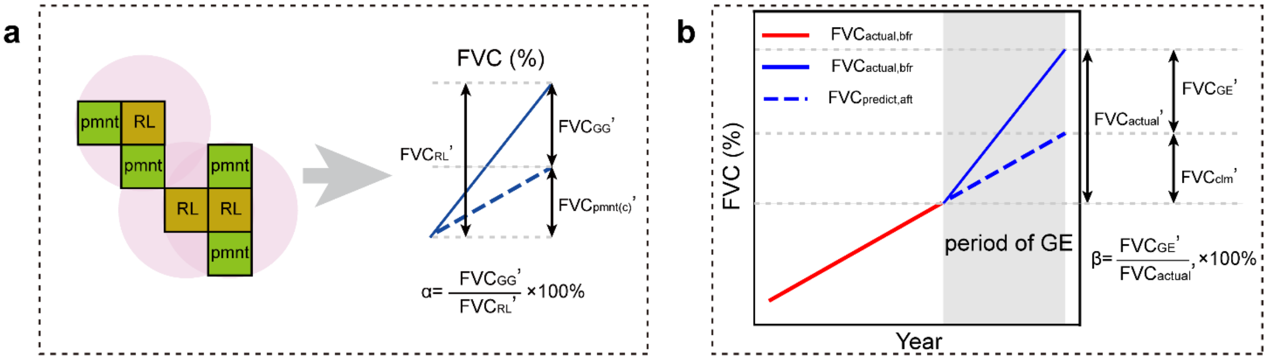

Supplementary Fig. 9 Diagram for estimating the contribution of "grain-for-green" (a) and grazing exclusion (b) practices to vegetation changes. a: Rehabilitated land (RL) and the adjacent permanent land use types (pmnt(c)) connected via buffers (pink circles), have similar climate, topographic and soil characteristics owing to the short distances among these areas. In the absence of "grain-for-green" practices, RL and pmnt(c) should show similar changes in fractional vegetation cover (FVC). The FVC trends before and after the grazing exclusion (GE) are shown in panel (b). The difference between the predicted FVC (FVCpredict, aft) driven by unknown factors (residuals) other than climate change and grazing exclusion practices and the actual FVC (FVCactual, bfr) thus comes only from the interference of grazing-prohibition activities due to the elimination of the effects of other land-use changes.

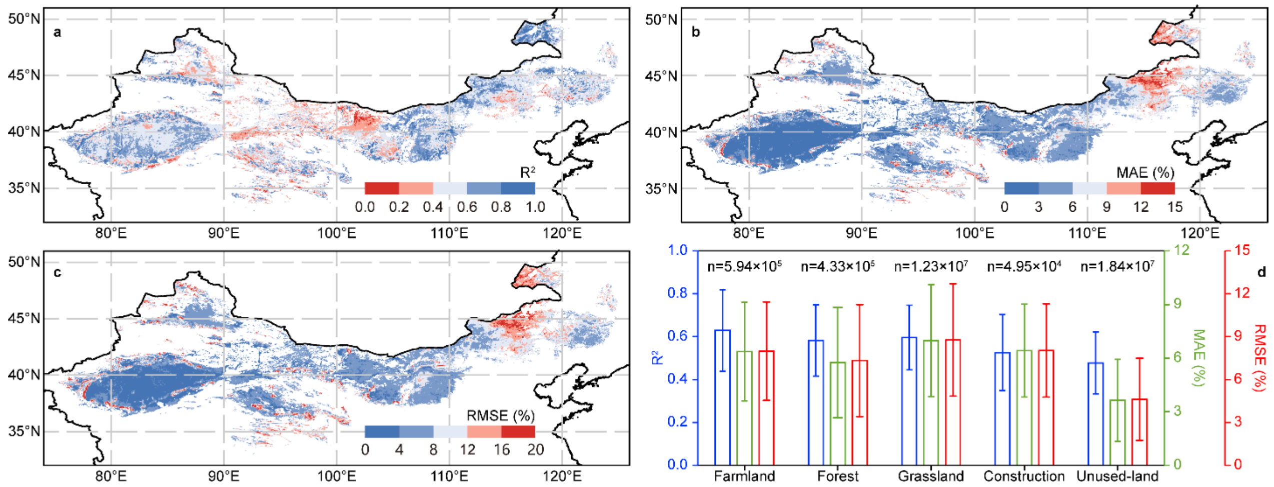

Supplementary Fig. 10 Stepwise multiple linear regression model error. Panels (a-c) show the spatial distribution of the coefficient of determination (R^2), the mean absolute error (MAE; %), and the root mean square error (RMSE; %), respectively. The bar chart in panel (d) represent the mean + /- standard deviation of R^2 (marked in blue), MAE (marked in green), and RMSE (marked in red) for each land use type, and statistical pixel sample sizes for farmland, forest, grassland, construction and unused-land are 5.94×10^5, 4.33×10^5, 1.23×10^7, 4.95×10^4, and 1.81×10^7, respectively. See Supplementary Fig. 6 for more details about land use spatial pattern.

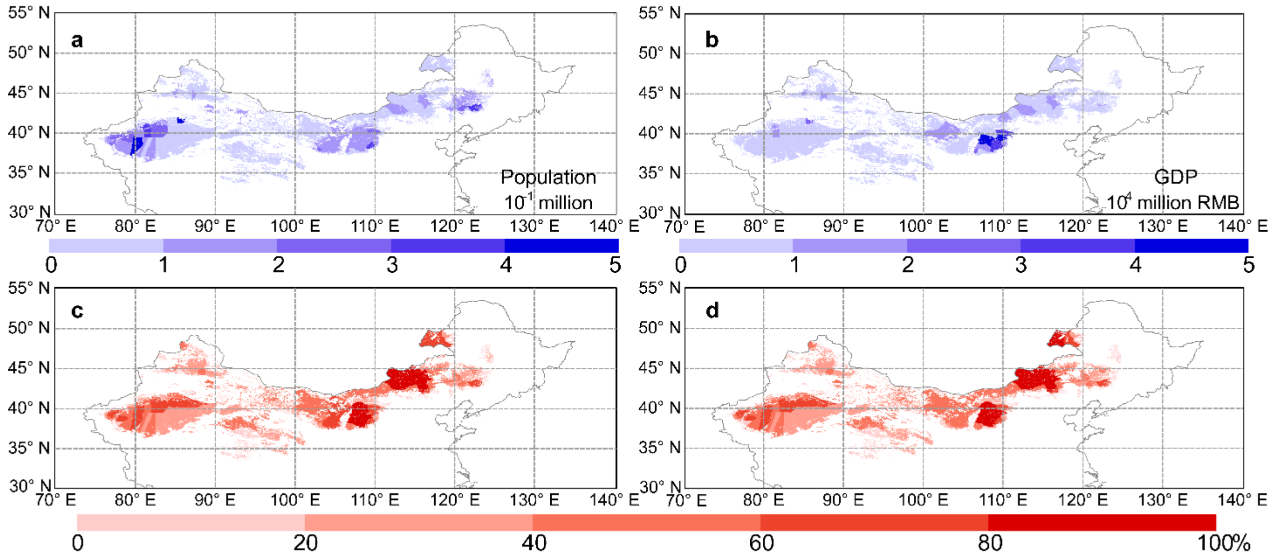

Supplementary Fig. 11 County-level population size (a), Gross Domestic Product (GDP, b), and their corresponding proportions (c and d) from 2015 to 2019 in the desertification-prone region of China.

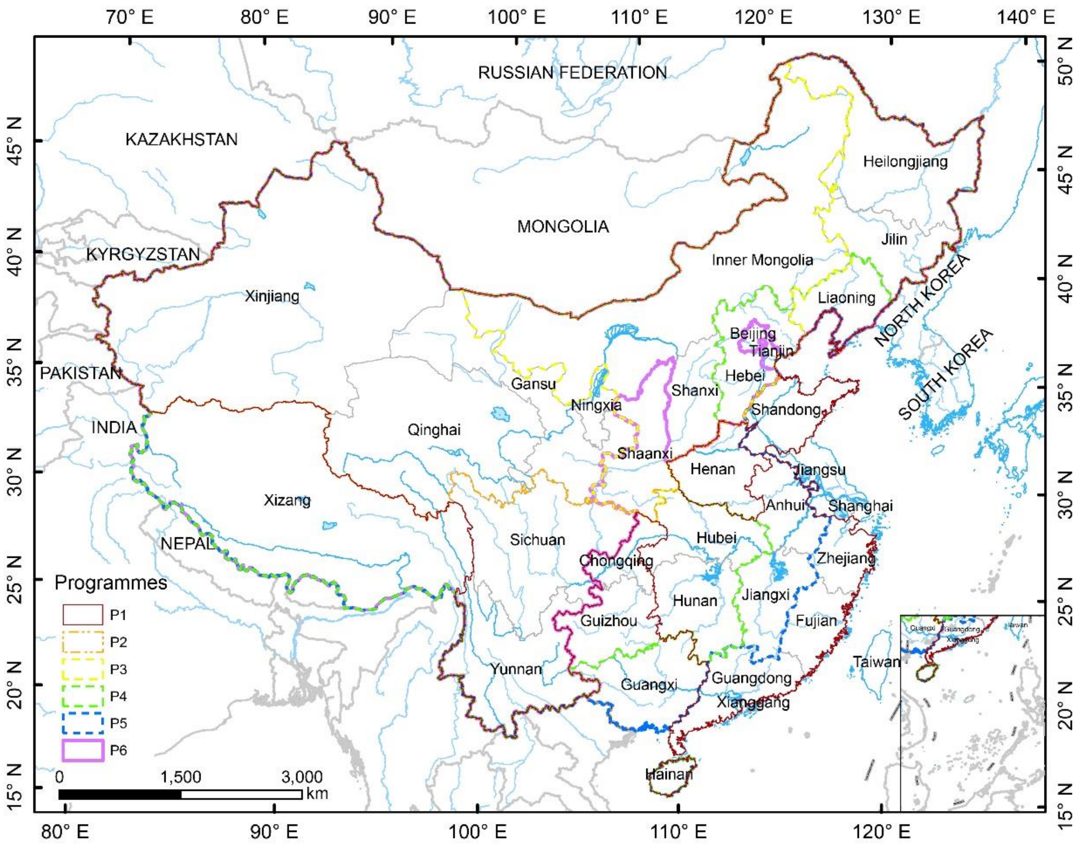

Supplementary Fig. 12 Locations of the provinces of China covered by the programs mentioned in Supplementary Table 11.

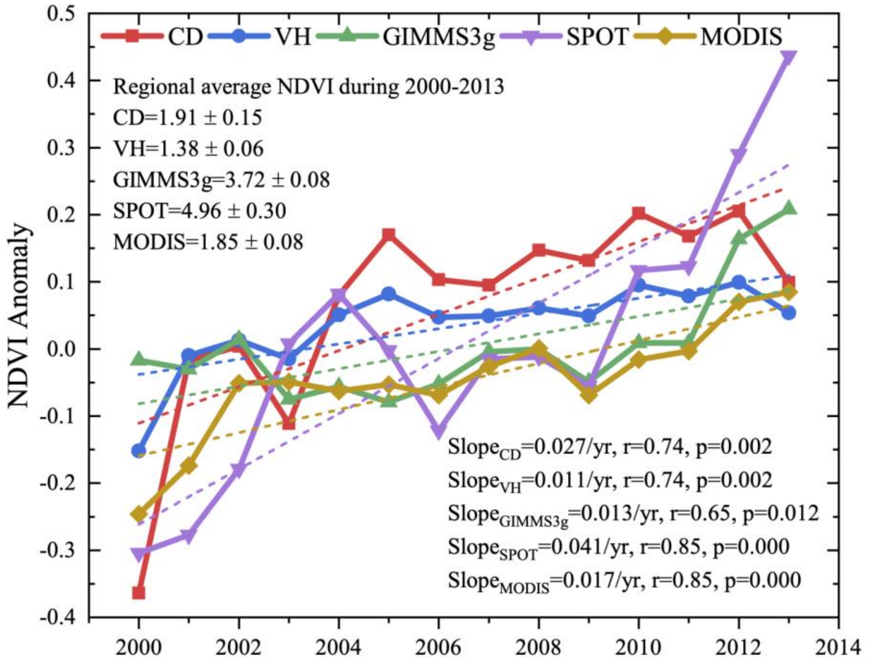

Supplementary Fig. 13 Comparison of the annual average the constructed (CD), the Blended Vegetation Health (VH), Global Inventory Modeling and Mapping Studies (GIMMS3g), Satellite Pour l'Observation de la Terre (SPOT), and Moderate-resolution Imaging Spectroradiometer (MODIS) normalized difference vegetation index (NDVI) anomalies in the desertification-prone region in China from 2000-2013. The regional average NDVI is relative to the multiyear mean ±1 standard deviation. The slope refers to the linear change in slope. All statistical degrees of freedom are 13, and the sample sizes are all 14. The r value is Pearson's R.

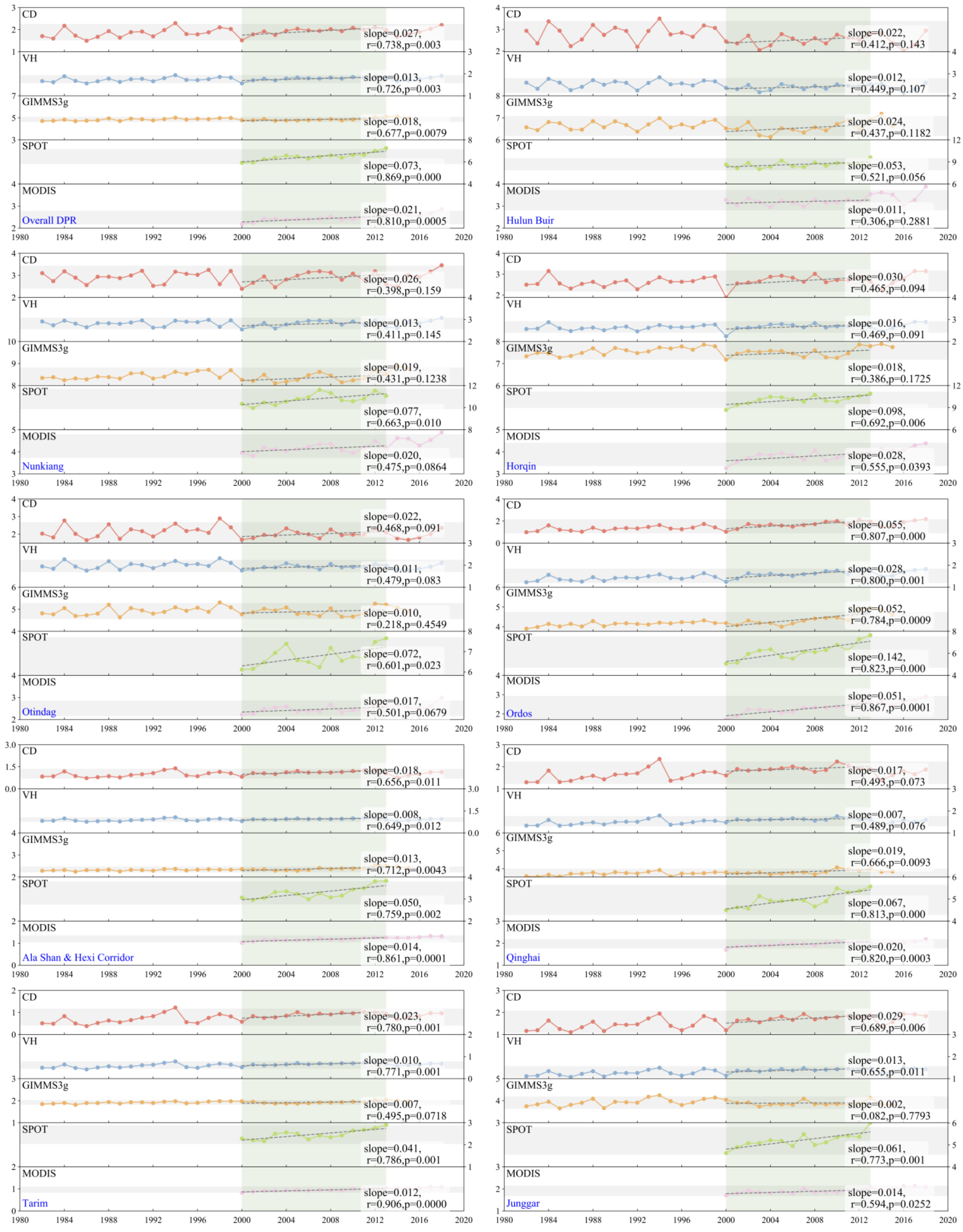

Supplementary Fig. 14 Annual variations in the constructed (CD), the Blended Vegetation Health (VH), Global Inventory Modeling and Mapping Studies (GIMMS3g), Satellite Pour l'Observation de la Terre (SPOT), and Moderate-resolution Imaging Spectroradiometer (MODIS) normalized difference vegetation index (NDVI) in the whole desertification-prone region and in each subregion. The locations of subregions are shown in Supplementary Fig. 2. The NDVI value is the sum of the values obtained for the 12 months in each year. The grey shading indicates NDVI ± 1.96 standard deviation (NDVI is the multiyear mean), and the green shading corresponds to the 2000-2013 period. All statistical degrees of freedom are 13, and the sample sizes are all 14. The r value is Pearson's R. The five coloured curves, from top to bottom, correspond to the CD, VH, GIMMS3g, SPOT,

and MODIS NDVI, respectively.

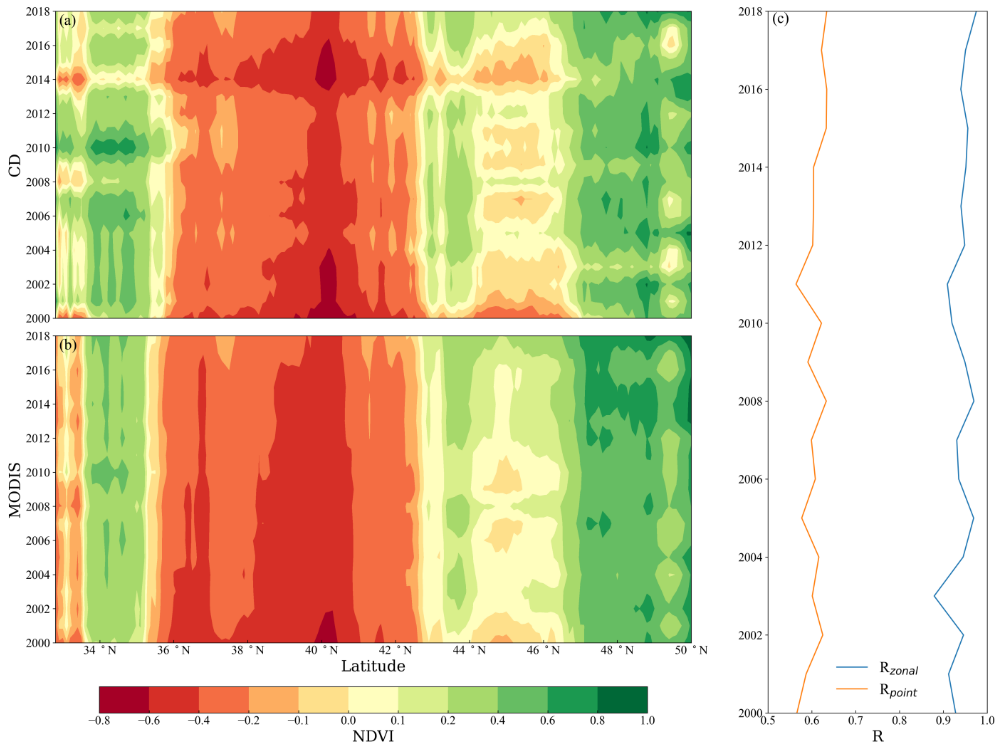

Supplementary Fig. 15 Zonal distribution of the constructed (CD) and Moderate-resolution Imaging Spectroradiometer (MODIS) 250-m normalized difference vegetation index (NDVI) series from 2000-2018 and annual variation of R_{zonal} and R_{point}. R_{zonal} is the correlation coefficient between the zonal CD and MODIS 250-m NDVI, and R_{point} is the point-to-point (or pixel-to-pixel) correlation coefficient between the CD and MODIS 250-m NDVI in the DPR. R_{zonal} and R_{point} are significant at the 99% confidence level (the minimum R values are 0.879 and 0.564, respectively, and the average sample sizes are 172 and 29,572,563, respectively). The anomaly was calculated as follows $(NDVI-NDVI_{mean})/NDVI_{mean}$. Where $NDVI_{mean}$ is the average value of all zonal-mean NDVI values from 2000 to 2018.

Supplementary Tables 1~13

Supplementary Table 1 Current major desertification-combating programmes launched in the desertification-prone region of China.

Programme	Year	Category †	Area covered ($\times10^4$ km^2)			Investment amount (billions of RMB) ‡			Data source
			A1	A2	A3	Capital investment	Central Govt budget	Total investment	
P1 §	1991-2000	GG/GE	1.49	2.60	0.49	1.98	0.67	3.42	Ref.[46]
	2005-2010	—	—	—	—	—	—	—	—
	2011-2020	—	—	—	—	—	—	—	—

P2		1978-present	GG/GE	37.87	12.46	—	66.61	34.83	278.12 ¶	Ref.[47-50]
P3 ††		2002-present	GG/GE	8.01	1.73	2.53	67.64	47.68	68.71	Ref.[47,49]
P4		1998-present	GG/GE	6.17	2.73	—	235.25	218.84	235.28	Ref. [47,49]
P5		1999-present	GG	15.85	0.90	2.07	240.48	219.79	240.84	Ref. [47,49]
P6	1 ‡‡	2002-present	GE	—	—	78.12				Ref.[47,50-52]
	2	2002-present	GE	—	—	105.16	29.07	29.07	29.07	
	3	2002-present	GE	—	—	19.03				
	4	2011-present	GE	—	—	170.00	145.91	145.91	145.91	
Total			—	69.39	20.42	441.66	786.94	696.79	1001.36	—

Note: A1: Afforestation; **A2**: mobile sands/hillside anchoring; **A3**: grazing prohibition/fence provision/grass-planting/grassland improvement.

P1: National Sand Control Programme, aiming to combat aeolian desertification by mobile sand anchoring, afforestation, grassland management, water-saving irrigation, and ecological resettlement; **P2**: Great Green Wall Programme, aiming to anchor mobile sands and to control dust storms by afforestation; **P3**: Beijing-Tianjin Sandstorm Source Control Programme, aiming to control dust storms occurred in Beijing and Tianjin by afforestation and by returning farmland to grassland; **P4**: Natural Forest Protection Programme, afforestation and controlling deforestation; **P5**: Returning Farmland to Forests/Grassland Programme; **P6**: National Grassland Ecological Protection and Construction Programme, aiming to mitigate grassland degradation by grazing exclusion (**P6-1**), grassland fencing and closure (**P6-2**), grassland management (**P6-3**), and ecological compensation (**P6-4**).

† GG and GE represent "grain-for-green" and grazing exclusion practices, respectively, indicating the main countermeasures of these programme. ‡ Investment amounts were converted to the equivalent amount in 2019 according to the inflation rate calculated by the consumer price index (CPI; CPI data were obtained from the National Bureau of Statistics of the PRC, http://www.stats.gov.cn/), $V_t=V_0*\Pi(1+CPI_i)$, i=0, 1, …, t-1, and the unconverted capital investment was ~650 billion RMB (~93 USD). § Statistics for programme P1 were available only for the first phase of the programme. ¶ The total investment was calculated as the sum of direct capital investments converted from labour hours. The labour hours before 1997 for the Great Green Wall Programme were obtained from ref.[49], and the allocation scheme was obtained from ref.[47]. †† Data for the Beijing-Tianjin Sandstorm Source Control Programme, also including the data before 2001. ‡‡ Grazing exclusion was practised in 2002. After 2011, the new policies of the Chinese government mainly included ecological compensation practices; among such policies, the compensation for grazing exclusion was 112.5 RMB/ha and that for grazing limitation was 37.5 RMB/ha[53].

Supplementary Table 2 Planned programmes related to combating desertification in the near future in China.

Programme	Period	Targeted investment (RMB)	Covered regions	Programme objectives	Data source
P1	2021-2050	—	1	1	Ref.[54]
P2	2013-2022	~87.8 billion	2	2	Ref.[55]
P3	2014-2020	~63.6 billion	3	3	Ref.[56]
P4	2016-2030	—	4	4	Ref.[57,58]
P5	2016-2025	—	5	5	Ref.[57,59]

P6	1	2016-2020	—	6	6	Ref.[60]
	2	2013-2022	~87.8 billion	2		
	3	2014-2020	~63.6 billion	3		
	4	2016-2020	18.7 billion/Year	7		
P7		2014-2020	Quota	8	7	Ref.[52]

Note：P1: Phase III of the Great Green Wall Programme; **P2:** Phase II of the Beijing-Tianjin Sandstorm Source Control Programme; **P3:** New round of the Returning Farmland to Forests/Grasslands Programme; **P4:** Sand Control Programme along the 'Silk Road'; **P5:** National Desert Park Programme; **P6:** The 13th Five-Year Plan for national grassland protection and use; **P6-1:** Pasturing prohibition; **P6-2:** Phase II of the Beijing-Tianjin Sandstorm Source Control Programme; **P6-3:** New round of the Returning Farmland to Forests/Grasslands Programme; **P6-4:** Compensation for grassland ecological conservation; **P7:** Aerial seeding programme for reclaimed grassland in the arable-pastoral zone.

Covered regions: 1, Northern China (4.36 million km^2); **2,** Beijing, Tianjing, Hebei, Inner Mongolia, Shanxi, and Shaanxi Provinces. **3,** 2.83 million ha in 18 provinces (Western China First); **4,** Nations and regions along the "Silk Road"; **5,** 17 Provinces in northern China (including 440 counties); **6,** The whole of China; **7,** 13 provinces in northern China; and **8,** 6 provinces in northern China (including 138 counties).

Programme objectives: 1, controlling sand and soil erosion; **2,** combating desertification and controlling dust storms; **3,** returning farmlands to forests/grasslands; **4,** revegetation; **5,** protecting wildlife resources, restoring desertified lands and promoting revegetation to achieve sustainable development by establishing national desert parks; **6,** managing grasslands by restoring vegetation cover, and increasing productivity, and improving herders' income; and **7,** returning farmlands to grasslands.

Supplementary Table 3 Historical fractional vegetation cover (FVC) trend statistics for different land uses. slopeMean: average FVC trend at the pixel scale (% a^{-1}); sigSlopeMean: average FVC significant trend (passes the Mann-Kendall test at the 95% significance level, same below) at the pixel scale (% a^{-1}); incArea: area with increasing FVC trends (km^2); decArea: area with decreasing FVC trends (km^2); sigIncArea: area with significantly increasing FVC trends (km^2); and sigDecArea: area with significantly decreasing FVC trends (km^2). Because grazing exclusion has been launched since 2003 in some areas of the desertification-prone region, the trends over the 2003-2018 period were also examined.

	From 1982 to 2018					
	slopeMean	sigSlopeMean	incArea	decArea	sigIncArea	sigDecArea
Farmland	1.12	0.84	14960.99	3880.27	12314.40	1652.35
Forest	0.46	0.29	9112.42	6648.09	6740.11	3376.23
Grassland	0.25	0.07	207011.30	246511.30	112688.83	83332.76
	From 2003 to 2018					
	slopeMean	sigSlopeMean	incArea	decArea	sigIncArea	sigDecArea
Farmland	1.15	2.36	14058.62	4696.89	7508.43	722.11
Forest	0.34	0.97	8683.15	7030.40	3105.28	1443.07
Grassland	0.05	0.46	199993.63	252071.02	42667.82	42222.46

Supplementary Table 4 Expected and actual production (tonnes) of grain and meat without the restrictions on available lands put in place by the "grain-for-green" (GG) and grazing exclusion (GE) practices in the desertification-prone region

and according to the corresponding reductions (%) in production and income. In accordance with the times that these two practices were extensively launched, grain and meat production losses were estimated since 2001 and 2011, respectively.

Period	Grain production involving GG			Meat production involving GE			Income cost
	Expected production	Actual production	Production loss	Expected production	Actual production	Production loss	
2001	11879640	10163032		-	-	-	
2002	13467509	11521454		-	-	-	
2003	14377161	12299661	14.45	-	-	-	9.14
2004	15201598	13004967		-	-	-	
2005	16505636	14120572		-	-	-	
2006	16218971	14009947		-	-	-	
2007	16687038	14414263		-	-	-	
2008	17769063	15348917	13.62	-	-	-	7.92
2009	17158871	14821833		-	-	-	
2010	18336576	15839134		-	-	-	
2011	18856231	16749990		2051191	1539624		
2012	20274297	18009658		2058155	1544851		
2013	22508206	19994039	11.17	2127502	1596903	24.94	17.72
2014	23337900	20731057		2223874	1669240		
2015	24359588	21638622		2237978	1679826		
2016	25128342	21482220		2181780	1669062		
2017	25469389	21773781		2119807	1621652		
2018	26574764	22718766	14.51	2057455	1573953	23.5	18.77
2019	27645832	23634422		2123305	1624328		
2020	28017946	23952542		2133431	1632075		

Supplementary Table 5 Population, the gross domestic product (GDP), and the income of arable-land farmers and livestock herders in the desertification-prone region. The conversion coefficient is based on the consumer price index (CPI) (http://tongji.cnki.net/kns55/Navi/NaviDefault.aspx), and GDP and incomes are converted to 2020 equivalent amounts. SD is the standard deviation.

Year	2015	2016	2017	2018	2019	2015~2019 Mean	2015~2019 SD
* Conversion coefficient	1.12	1.09	1.08	1.05	1.03	-	-
Population ($\times 10^4$)	1529.25	1532.75	1530.13	1537.14	1537.58	1533.37	3.87
GDP ($\times 10^8$ RMB)	10682.24	10546.85	10474.37	11105.78	11783.23	10918.49	541.76
Disposable income of arable farmers and livestock herders ($\times 10^8$ RMB)	1810.336	1902.752	2052.064	2199.929	2334.124	2059.84	213.22
Income from arable agriculture and animal husbandry ($\times 10^8$ RMB)	954.40	899.96	923.44	978.38	920.20	935.28	30.98

Supplementary Table 6 Direct payments from the Chinese authorities to arable farmers and livestock herders related to

“grain-for-green” and grazing exclusion practices. The total sum from 1999 to 2004 was 100 RMB ≈ 14 USD. The yearly average payments of the two practices in the most recent 5 years (2014-2018) were ~6.82 billion RMB and 17.86 billion RMB at the provincial scale, respectively. According to the area ratio of the “grain-for-green” (9.72%) and grazing exclusion (28.23%) practices located in the desertification-prone region (DPR), the yearly average payments of these two practices reached ~0.66 billion RMB and ~5.04 billion RMB, respectively, and the yearly total payment was ~5.70 billion RMB (~0.80 billion USD). The areas of returned farmland for “grain-for-green” practices in the DPR and at the provincial scale (11 provinces) were 5634.5 km^2 and 57,981.36 km^2, respectively, based on statistics from land-use changes from 2000 to 2020. The areas of grasslands involved in grazing exclusion practices in the DPR and nationwide were 48 million km^2 and ~170 million km^2, respectively. These data were obtained from the Editorial Department of China Environmental Yearbook (2003-2018) and China Forestry Statistical Yearbook (1998-2017).

Year	Grain-for-green (100 million RMB)			Grassland ecological compensation (100 million RMB)
	Living subsidy	Grain subsidy	Direct payment	
*1999~2004	61.61	77.41	139.02	/
2005	14.37	104.38	118.75	/
2006	14.59	100.40	114.99	/
2007	15.33	109.05	124.38	/
2008	15.42	107.52	122.94	/
2009	15.83	102.60	118.42	/
2010	22.60	80.27	102.87	/
2011	19.98	60.67	80.64	136.00
2012	20.10	47.65	67.75	150.00
2013	22.35	30.63	52.97	159.46
2014	54.71	2.22	56.93	160.69
2015	52.48	14.81	67.29	169.49
2016	51.16	19.36	70.52	187.60
2017	47.85	23.09	70.93	187.60
2018	35.64	39.41	75.05	187.60
2014~2018 ave.	48.37	19.78	68.15	178.60

Supplementary Table 7 Future fractional vegetation cover (FVC) trend statistics for different land uses under different shared socioeconomic pathway (SSP) and representative concentration pathway (RCP) scenarios. The meaning and units of the indicator are the same as those described in Supplementary Table 3.

	From 2015 to 2050 in SSP1-2.6					
	slopeMean	sigSlopeMean	incArea	decArea	sigIncArea	sigDecArea
Farmland	1.06	1.09	13726.38	2454.46	13496.16	2243.67
Forest	0.39	0.40	9824.90	5772.79	9502.21	5434.39
Grassland	0.08	0.09	204815.91	251512.83	190275.40	234110.80
	From 2015 to 2050 in SSP2-4.5					
	slopeMean	sigSlopeMean	incArea	decArea	sigIncArea	sigDecArea
Farmland	1.56	1.60	13235.03	2945.81	13052.54	2799.67
Forest	0.54	0.56	9525.79	6071.89	9293.40	5892.86

Grassland	0.04	0.04	188264.83	268063.90	180342.08	258837.80
	From 2015 to 2050 in SSP5-8.5					
	slopeMean	sigSlopeMean	incArea	decArea	sigIncArea	sigDecArea
Farmland	2.37	2.41	13085.63	3095.22	12972.37	2955.39
Forest	0.82	0.83	9470.05	6127.63	9330.73	5985.60
Grassland	0.03	0.03	185108.28	271220.45	180149.71	265774.80

Supplementary Table 8 Comparison of the averaged of the constructed (CD), the Blended Vegetation Health (VH), Global Inventory Modeling and Mapping Studies (GIMMS3g), Satellite Pour l'Observation de la Terre (SPOT), and Moderate-resolution Imaging Spectroradiometer (MODIS) fractional vegetation cover values in different regions in the desertification-prone region from 2000-2013. The locations of these regions are shown in Supplementary Fig. 2.

Region	CD	VH	GIMMS3g	SPOT	MODIS	Mean	SD (Standard Deviation)
Hulun Buir	0.606	0.725	0.681	0.694	0.471	0.635	0.102
Nunkiang	0.703	0.853	0.875	0.863	0.64	0.787	0.108
Horqin	0.664	0.829	0.805	0.821	0.574	0.739	0.114
Otindag	0.471	0.571	0.486	0.514	0.338	0.476	0.086
Ordos	0.376	0.461	0.425	0.469	0.299	0.406	0.070
Ala Shan & Hexi Corridor	0.208	0.231	0.174	0.193	0.110	0.183	0.046
Qinghai	0.428	0.456	0.353	0.35	0.251	0.368	0.080
Tarim	0.145	0.135	0.125	0.124	0.076	0.121	0.027
Junggar	0.354	0.381	0.365	0.373	0.238	0.342	0.059
Over DPR	0.439	0.516	0.476	0.489	0.333	0.451	0.071

Supplementary Table 9 Pearson's correlation coefficients between the constructed (CD), the Blended Vegetation Health (VH), Global Inventory Modeling and Mapping Studies (GIMMS3g), Satellite Pour l'Observation de la Terre (SPOT), and Moderate-resolution Imaging Spectroradiometer (MODIS) fractional vegetation cover in different regions in the desertification-prone region from 2000-2013. The locations of these regions are shown in Supplementary Fig. 2. The '*' symbol indicates that the correlation coefficient is significant at the 95% confidence level; the '**' symbol indicates that the correlation coefficient is significant at the 99% confidence level.

Region	VH	GIMMS3g	SPOT	MODIS
Hulun Buir	0.97^{**}	0.81^{**}	0.84^{**}	0.64^{*}
Nunkiang	0.98^{**}	0.52	0.66^{**}	0.64^{*}
Horqin	1.00^{**}	0.56^{*}	0.85^{**}	0.89^{**}
Otindag	1.00^{**}	0.69^{**}	0.86^{**}	0.85^{**}
Ordos	1.00^{**}	0.74^{**}	0.88^{**}	0.93^{**}
Ala Shan & Hexi Corridor	0.99^{**}	0.31	0.45	0.67^{**}
Qinghai	1.00^{**}	0.60^{*}	0.68^{**}	0.81^{**}
Tarim	0.99^{**}	0.13	0.57^{*}	0.69^{**}
Junggar	1.00^{**}	0.05	0.81^{**}	0.78^{**}

Supplementary Table 10 Commonly used global climate models (GCMs) in the Coupled Model Intercomparison Project Phase 6 (CMIP6) model experiments[61].

No.	GCM	Grid (lon × lat)	Organization
1	ACCESS-ESM1-5	192×145	CSIRO, Australia
2	CanESM5	128×64	CCCma, Canada
3	CanESM5-CanOE	128×64	CCCma, Canada
4	CAS-ESM2-0	256×128	CAS, China
5	CMCC-CM2-SR5	288×192	CMCC, Italy
6	CMCC-ESM2	288×192	CMCC, Italy
7	CNRM-CM6-1	256×128	CNRM-CERFACS, France
8	CNRM-ESM2-1	256×128	CNRM-CERFACS, France
9	EC-Earth3-Veg	512×256	EC-Earth-Consortium
10	FGOALS-g3	180×80	CAS, China
11	FIO-ESM-2-0	288×192	FIO-QLNM, China
12	GFDL-ESM4	288×180	NOAA-GFDL, US
13	GISS-E2-1-G	144×90	NASA-GISS, US
14	HadGEM3-GC31-LL	192×144	MOHC, NERC, UK
15	INM-CM4-8	180×120	INM, Russia
16	INM-CM5-0	180×120	INM, Russia
17	IPSL-CM6A-LR	144×143	IPSL, France
18	MIROC-ES2L	128×64	MIROC, Japan
19	MPI-ESM1-2-HR	384×192	MPI-M, DWD, DKRZ, Germany
20	MPI-ESM1-2-LR	192×96	MPI-M, AWI, DKRZ, DWD, Germany
21	UKESM1-0-LL	192×144	MOHC, NERC, NIMS-KMA, NIWA

Supplementary Table 11 Detailed countermeasures of ecological programmes in China.

Programme	Provinces included	Countermeasure	Land use type	References
**P1**	BJ, TJ, HE, SX, NM, LN, JL, HLJ, ZJ, AH, FJ, JX, SD, HN, HB, GD, HI, SC, YN, SN, GS, NX, QH, XJ	(1) Afforestation, (2) Closing hillsides to facilitate afforestation and sandy land protection, (3) Artificial glass planting in grassland, (4) Aeolian desertified land (grassland and farmland included) controlling, (5) Mobile sand anchoring, (6) Water-saving irrigation, and (7) Ecological resettlement	Forest, grassland, and farmland	Ref.[62]
**P2**	BJ, TJ, HE, SX, NM, LN, JL, HLJ, SN, GS, NX, QH, XJ	(1) Afforestation, (2) Closing hillsides to facilitate afforestation and sandy land protection, (3) Tree planting along the sides of roads, ditches, canals, and houses, (4) Forest network building for farmlands, oases, and pasture, and (5) Grass / tree planting on surfaces of mobile and semi-anchored dunes	Forest, grassland, farmland, and unused land	Ref.[63,64]
**P3**	BJ, TJ, HB, SX, NM, SN	(1) Grain for green, (2) Afforestation, (3) Grassland construction, (4) Water controlling, and (5) Ecological resettlement	Forest, grassland, desertified farmland, and sandy land	Ref.[64,65]
**P4**	NM, HLJ, JL, HI, CQ, SC, GZ, YN,	(1) Deforestation forbidding in the upper and middle reaches of Yellow River and in the upper reaches of Yangtze River,	Forest	Ref.[64,66]

	HB, XZ, SX, SN, GS, NX, QH, XJ, HN	(2) Reducing timber harvesting in HLJ, JL, LN, NM, HN, and XJ, (3) Forest protection, and (4) Forest worker re-employment		
**P5**	HLJ, JL, LN, NM, BJ, TJ, HE, HA, AH, HB, HN, JX, HI, CQ, SC, GZ, YN, GX, SX, SN, GS, NX, QH, XJ, XZ	(1) Slope treatment for farmland and desertified sandy land (slope > 25°), (2) Afforestation in mountain, hill, and sandy land with sparse vegetation, and (3) Subsiding to farmers	Farmland and desertified sandy land (slope > 25°)	Ref.[64,66]
**P6**	NM, GS, NX, XJ, XZ, QH, SC, YN, HE, SX, LN, JL, HLJ	(1) Grassland fencing and enclosing, (2) Forage-livestock balance, (3) Rotation grazing, (4) Livestock shed feeding, and (5) Grazing exclusion, subsiding to farmers and herdsmen	Degraded grassland	Ref.[64,67,68]

Note：Programmes P1 to P6 are identified in Supplementary Table 1. The locations of the provinces of China are shown in Supplementary Fig. 12. Abbreviations of the provinces are established as follows: Beijing (BJ), Tianjin (TJ), Hebei (HE), Shanxi (SX), Inner Mongolia (NM), Liaoning (LN), Jilin (JL), Heilongjiang (HLJ), Shanghai (SH), Jiangsu (JS), Zhejiang (ZJ), Anhui (AH), Fujian (FJ), Jiangxi (JX), Shandong (SD), Henan (HA), Hubei (HB), Hunan (HN), Guangdong (GD), Guangxi (GX), Hainan (HI), Chongqing (CQ), Sichuan (SC), Guizhou (GZ), Yunnan (YN), Xizang (XZ), Shaanxi (SN), Gansu (GS), Qinghai (QH), Ningxia (NX), and Xinjiang (XJ).

Supplementary Table 12 Remote sensing interpretation accuracy analysis table, taking Northeast China as an example.

Land use types	Number of validation samples	Number of samples at different levels											
		L0	L1	L2	L3	L4	L5	L6	L7	L8	L9	L10	Overall accuracy (%)
Paddy field	62	7			1		2		1	3	1	47	84.35
Dryland	1463	81	0	4	3	1	17	6	3	21	16	1311	92.86
Woodland	1100	135	2	6	2	9	38	1	4	33	10	860	83.95
Shrubland	520	30		2	1	1	3			5	2	476	93.15
Sparse forest land	410	34					5	2	2	4	6	357	90.41
Other forest land	19	3				1	1		1	1		12	75.79
High coverage grassland	1039	71		3	5	4	19	6	3	15	11	902	90.74
Medium coverage grassland	606	27	1	1	1	5	11	3		7	2	548	93.28
Low-coverage grassland	52	2								1		49	95.77
River	27	1										26	96.3
Lake	421	23					4			3	2	389	93.87
Reservoir	22	2					1					19	88.64
Flood land	152	10					2					140	92.76
Urban	9											9	100
Rural	2030	14	1	1	2	3	9	3	1	11	7	1978	98.63
Industrial and mining land	5											5	100
Desert	16											16	100
Saline and alkaline land	325	60		6	3	1	16	6	1	10	1	221	75.29
Swamp	465	32	1				6			6	1	419	92.02
Bare land	10	8										2	20

Summary	8753	540	5	23	18	25	134	27	16	120	59	7786	88.95

Supplementary Table 13 Fitting and cross-validation results obtained for cultivated lands and grasslands in 18 counties in different areas of the desertification-prone region in 1995, 2005, and 2015. R^2 indicates the goodness of fit, and '**' indicates that the results are significant at the 99% confidence level. The cross-validation results show the error percentage between the observed and the predicted values in the corresponding year. The geographical locations of the counties listed in the table are shown in Supplementary Fig. 6. The region codes are established as follows: A1: Hulunbuir, A2: Nenjiang, A3: Horqin, A4: Hunshandak, A5: Ordos, A6: Alashan and Hexi Corridor, A7: Qinghai, A8: Tarim, and A9: Junggar.

Zone	County	Cropland area					Grassland area				
		Significance	R^2	Cross-validation results (%)			Significance	R^2	Cross-validation results (%)		
				1995	2005	2015			1995	2005	2015
A1	Xin Barag Youqi	**	0.96	4.42	25.98	-4.20	**	0.97	-0.05	-0.20	0.23
	Chen Barag Youqi	**	0.86	0.00	0.00	0.00	**	0.92	0.00	0.00	0.00
A2	Gannan	**	0.97	-6.33	-6.49	7.85	**	0.81	-2.39	0.37	2.67
	Tailai	**	0.96	-7.58	-0.32	-6.85	**	0.93	-6.04	-0.02	1.44
A3	Tongyu	**	0.93	-13.63	-3.30	10.37	**	0.92	35.91	6.60	-26.40
	Naiman	**	0.54	20.48	-7.18	-6.37	**	0.95	0.26	-1.06	0.39
A4	Abaga Qi	**	0.93	15.98	-34.90	3.46	**	0.92	-0.22	0.30	-0.06
	Sunite Youqi	**	0.82	30.96	-30.62	52.27	**	0.42	0.00	0.26	0.11
A5	Hangjin Qi	**	0.94	12.58	-11.42	-4.72	**	0.47	-0.18	2.05	-1.66
	Uxin Qi	**	0.90	-4.58	-24.11	-14.43	**	0.93	-4.20	6.51	-0.46
A6	Ala Shan Zuoqi	**	0.93	-0.65	2.12	-6.15	**	0.94	3.51	3.70	-8.24
	Ejin Qi	**	0.84	0.68	-1.36	-0.89	**	0.55	0.10	1.02	-2.84
A7	Dulan	**	0.80	10.15	0.35	-3.93	**	0.84	-0.09	-0.46	0.60
	Guinan	**	0.94	5.74	24.42	-7.88	**	0.97	-0.29	-0.42	0.16
A8	Yuli	**	0.98	8.84	4.85	3.86	**	0.96	9.20	2.54	-6.43
	Qira	**	0.95	11.58	36.72	-6.00	**	0.74	-5.39	0.07	5.36
A9	Fuhai	**	0.97	14.62	-10.91	-11.04	**	0.86	-3.24	-0.07	8.42
	Hutubi	**	0.96	14.76	11.20	-9.78	**	0.88	-2.29	-2.45	12.18

References:

1. UN Secretariat of the Conference on Desertification (UNCD). *Desertification: An overview, In: Desertification: Its Causes and Consequences*. (Pergamon Press, 1977).
2. United Nations. *Results of the World Conference on Environment and Development: Agenda 21. UNCED United Nations*

Conference on Environment and Development. Rio de Janerio. (1992).
3. The Central Government of the People's Republic of China. *The Third Communiqué on Desertification and Desertification in China.* http://www.gov.cn/ztzl/fszs/content_650487.htm (2007).
4. State Forestry Administration of China (SFAC). *The Bulletin of Status Quo of Desertification and Sandification in China*. http://www.forestry.gov.cn/ (2000, 2005, 2010, 2015).
5. Zhang, K., Xie, Y. & Wei, X. *Evaluation of Soil Erosion on the Loess Plateau*. (Science Press, 2015).
6. Cheng, G. *et al.* Characteristic, changes and impacts of permafrost on Qinghai-Tibet Plateau. *Chinese Sci. Bull.* **64**, 2783–2795 (2019).
7. Chang, Q. & Xie, B. *Vegetation of Dynamics and Climate Change of the Loess Plateau*. (Science Press, 2019).
8. Zhang, T., Huang, Y. & Yang, X. Climate warming over the past three decades has shortened rice growth duration in China and cultivar shifts have further accelerated the process for late rice. *Global Change Biol.* **19**, 563–570 (2013).
9. Zhou, H., Chen, Y. & Li, W. Effect of oasis hydrological processes on soil salinization of Tikanlik Oasis in the lower Tarim River. *Acta Geographica Sinica* **63**, 714–724 (2008).
10. Currell, M., Han, D., Chen, Z. & Cartwright, I. Sustainability of groundwater usage in northern China: dependence on palaeowaters and effects on water quality, quantity and ecosystem health. *Hydrol. Process.* **26**, 4050–4066 (2012).
11. Wang, T. *et al.* Contrasting groundwater depletion patterns induced by anthropogenic and climate-driven factors on Alxa Plateau, northwestern China. *J. Hydrol.* **576**, 262–272 (2019).
12. Xia, J. *et al.* Evaluating the dynamics of groundwater depletion for an arid land in the Tarim Basin, China. *Water* **11**, 186 (2019).
13. Zhu, Z. Current Situation and Prospec of Land Desertification Problem. *Geogr. Res.* **13**, 105–113 (1994).
14. Zhu, Z. Concept, Cause and Control of Desertification in China. *Quaternary Sciences* **18**, 145–155 (1998).
15. Zhu, Z. & Chen, G. *Sandy Desertification in China*. (Science Press, 1994).
16. Reynolds, J. *et al.* Global Desertification: Building a Science for Dryland Development. *Science* **316**, 847–851 (2007).
17. Ci, L. & Yang, X. *Desertification and its control in China*. (Higher Education Press, 2010).
18. Cooke, R. U. Stone pavement in deserts. *Annals of the Association of American Geographers* **60**, 560–577 (1970).
19. Chen, X. *Physical Geography of Arid land in China*. (Science Press, 2000).
20. Zhu, Z. & Liu, S. *Desert Evolutions in Historical Periods of China. Physical Geography in China (Vol. Historical Geography)*. (Science Press, 1982).
21. Yin, P., Fang, X., Tian, Q. & Ma, Y. Distribution and regional difference of main output regions in grain production in China in the early 21st century. *Acta Geographica Sinica* **61**, 190–198 (2006).
22. Sun, Z., Du, Q., Han, C. & Min, Z. *General Agricultural History of China-The Ming and Qing Dynasties*. (China Agriculture Press, 2016).
23. Li, J., Li, B., Hu, P. & Tian, Y. *Economic History of Sheep in China*. (China Agricultural Science and Technology Press, 2015).
24. Wang, X. Sandy desertification: Borne on the wind. *Chinese Sci. Bull.* **58**, 2395–2403 (2013).
25. Zhou, Z., Zhu, Z. & Liu, Z. *Restoration of Degraded Arid Desert Ecosystems and Sustainable Development*. (Science Press, 2010).
26. Bao, G., Qin, Z., Bao, Y. & Zhou, Y. Spatial-temporal changes of vegetation cover in Mongolian plateau during 1982-2006. *Journal of Desert Research* **33**, 918–927 (2013).
27. Lu, H. *et al.* Formation and evolution of Gobi Desert in central and eastern Asia. *Earth-Sci. Rev.* **194**, 251-263 (2019).
28. Hird, J. & McDermid, G. Noise reduction of NDVI time series: An empirical comparison of selected techniques. *Remote Sens. Environ.* **113**, 248–258 (2009).
29. Ma, M. & Veroustraete, F. Reconstructing pathfinder AVHRR land NDVI time-series data for the Northwest of China. *Adv. Space Res.* **37**, 835–840 (2006).
30. Fensholt, R., Rasmussen, K., Nielsen, T. & Mbow, C. Evaluation of earth observation based long term vegetation trends - Intercomparing NDVI time series trend analysis consistency of Sahel from AVHRR GIMMS, Terra MODIS and SPOT VGT data. *Remote Sens. Environ.* **113**, 1886–1898 (2009).
31. Tian, F. *et al.* Evaluating temporal consistency of long-term global NDVI datasets for trend analysis. *Remote Sens. Environ.* **163**, 326–340 (2015).

32. Meinshausen, M. *et al*. The shared socio-economic pathway (SSP) greenhouse gas concentrations and their extensions to 2500. *Geoscientific Model Development* **13**, 3571–3605 (2020).
33. O'Neill, B.C. *et al*. The Scenario Model Intercomparison Project (ScenarioMIP) for CMIP6. *Geoscientific Model Development* **9**, 3461–3482 (2016).
34. Lalande, M., Ménégoz, M., Krinner, G., Naegeli, K. & Wunderle, S. Climate change in the High Mountain Asia in CMIP6. *Earth System Dynamics* **12**, 1061–1098 (2021).
35. Donohue, R.J., Roderick, M.L., McVicar, T.R. & Farquhar, G.D. Impact of CO_2 fertilization on maximum foliage cover across the globe's warm, arid environments. *Geophysical Research Letters* **40**, 3031–3035 (2013).
36. Piao, S. et al. Detection and attribution of vegetation greening trend in China over the last 30 years. *Global Change Biology* **21**, 1601–1609 (2015).
37. Meinshausen, M. *et al*. Historical greenhouse gas concentrations for climate modelling (CMIP6). *Geoscientific Model Development* **10**, 2057–2116 (2017).
38. Teutschbein, C. & Seibert, J. Bias correction of regional climate model simulations for hydrological climate-change impact studies: Review and evaluation of different methods. *Journal of Hydrology* **456–457**, 12–29 (2012).
39. Xu, X. *et al*. Remote Sensing Monitoring Data of Land Use in China. (Data Registration and Publishing System of the Resource and Environmental Science Data Center of the Chinese Academy of Sciences, 2018). https://www.resdc.cn/data.aspx?DATAID=335
40. Liu, J. *et al*. Spatiaotemporal characteristics, patterns and causes of land-use changes in China since the late 1980s. *J. Geogr. Sci.* **24**, 195–210 (2014).
41. Liu, J. *et al*. Spatio-temporal patterns and characteristics of land-use change in China during 2010-2015. *J. Geogr. Sci.* **73**, 789–802 (2018).
42. Li, J., Li, Z., Wu, H. & You, N. Trend, seasonality, and abrupt change detection method for land surface temperature time-series analysis: Evaluation and improvement. *Remote Sens. of Environ.* **280**, 113222 (2022).
43. Li, Y., L, Y., Westlund, H. & Liu, Y. Urban–rural transformation in relation to cultivated land conversion in China: Implications for optimizing land use and balanced regional development. *Land Use Policy* **47**, 218–224 (2015).
44. Hocking, R. R. Developments in Linear Regression Methodology: 1959-1982. *Technometrics* (1983).
45. Zhang, G., Zhang, Y., Dong, J. & Xiao, X. Green-up dates in the Tibetan Plateau have continuously advanced from 1982 to 2011. *Proc. Natl. Acad. Sci. USA* **110**, 4309–4314 (2013).
46. State Forestry Administration of the PRC (SFA). *China Forestry Statistical Yearbook 1998-2017*. (China Forestry Publishing House, 1998-2017).
47. Kang, R. The largest ecological project in China-Three North Shelterbelt System. *Environmental Protection* **3**, 6–8 (1988).
48. National Forestry and Grassland Administration of the PRC (NFGA). *China Forestry and Grassland Statistical Yearbook 2018*. (China Forestry Publishing House, 2018).
49. Sun, C. Historical review and perspective of investment and task in the construction of the three North Shelterbelt System. *Protection Forest Science and Technology* **91**, 59–61 (2009).
50. Bureau of Agriculture and Animal Husbandry of the PRC (BAAH). National plan for grassland ecological protection and construction from 2001 to 2010. *Pratacultural Science* **20**, 68–69, 80–84 (2003).
51. China News. *Suggestions on improving the policy of returning grazing land to grassland issued by the three ministries.* http://www.chinanews.com/cj/2011/08-31/3297565.shtml (2011).
52. National Development and Reform Commission of the PRC (NDRC). *Work plan for the construction of the pilot project of cultivated grassland management in agro-pastoral ecotone in 2016.* (2016).
53. Geng, G. Grassland ecological protection has a long way to go. *Green China* **15**, 30–33 (2018).
54. Zhu, J. & Zheng, X. The prospects of development of the Three-North Afforestation Program (TNAP): On the basis of the results of the 40-year construction general assessment of the TNAP. *Chinese Journal of Ecology* **38**, 1600–1610 (2019).
55. Central Government Website of the PRC. *Total investment of the second phase of the Beijing-Tianjin Sandstorm Source Control Project will reach 87.792 billion yuan.* http://www.gov.cn/jrzg/2012-10/07/content_2238556.htm (2012).
56. State Forestry Administration of the PRC (SFA). *The State Council approved the implementation of the general plan for a new round of the Grain for Green.* http://www.forestry.gov.cn/main/4093/content-730197.html (2015).

57. Bao, Y. *et al.* Review of 60 years combating desertification in China and prospects on it. *Science of Soil and Water Conservation* **16**, 144–150 (2018).
58. China Green Times. *Belt and Road Initiative to combat desertification.* http://www.forestry.gov.cn/main/72/content-881674.html (2016).
59. State Forestry Administration of the PRC (SFA). *China Forestry Yearbook 2016*. (China Forestry Publishing House, 2016).
60. Ministry of Agriculture of the PRC. *Circular of the Ministry of Agriculture on printing and distributing the 13th five-year plan for national grassland protection, construction, and utilization.* http://www.moa.gov.cn/nybgb/2017/dyiq/201712/t20171227_6129885.htm (2017).
61. Eyring, V. *et al*. Overview of the Coupled Model Intercomparison Project Phase 6 (CMIP6) experimental design and organization. *Geoscientific Model Development* **9**, 1937-1958 (2016).
62. China National People's Congress (NPC). Interpretation of the Law of the People's Republic of China on Prevention and Control of Desertification, Chapter II Planning for Prevention and Control of Desertification. http://www.npc.gov.cn/npc/c2181/flsyywd_list.shtml (2003).
63. Wang, X. M., Zhang, C. X., Hasi, E. & Dong, Z. B. Has the Three Norths Forest Shelterbelt Program solved the desertification and dust storm problems in arid and semiarid China? *Journal of Arid Environments* **74**, 13–22 (2010).
64. Bryan, B. A. *et al*. China's response to a national land-system sustainability emergency. *Nature* **559**, 193–204 (2018).
65. PRC National Development and Reform Commission (NDRC). Planning of Beijing-Tianjin Sandstorm Source Control Program (2001-2010). https://www.ndrc.gov.cn/fggz/fzzlgh/gjjzxgh/200709/t20070928_1196575_ext.html (2007).
66. Yin, R. & Yin, G. China's primary programs of terrestrial ecosystem restoration: initiation, implementation, and challenges. *Environ. Manage* **45**, 429–441 (2010).
67. Yin, Y., Hou, Y., Langford, C., Bai, H. & Hou, X. Herder stocking rate and household income under the Grassland Ecological Protection Award Policy in northern China. *Land Use Policy* **82**, 120–129 (2019).
68. Sun, J. *et al*. Reconsidering the efficiency of grazing exclusion using fences on the Tibetan Plateau. *Science Bulletin* **65**, 1405–1414 (2020).
